# Obesity and kidney protection

**DOI:** 10.12860/jnp.2014.18

**Published:** 2014-07-01

**Authors:** Aravind Chandra, Michael Biersmith, Ramin Tolouian

**Affiliations:** Division of Nephrology and Hypertension, Eastern Virginia Medical School, Norfolk, VA, USA

**Keywords:** Fat, Metabolic syndrome, Renal disease

## Abstract

*Context:* Obesity, both directly and indirectly, increases the risk for a variety of disease conditions including diabetes, hypertension, liver disease, and certain cancers, which in turn, decreases the overall lifespan in both men and women. Though the cardiovascular risks of obesity are widely acknowledged, less often identified is the relationship between obesity and renal function.

*Evidence Acquisitions:* Directory of Open Access Journals (DOAJ), Google Scholar, PubMed, EBSCO and Web of Science has been searched.

*Results:* The concept of the “Metabolic Syndrome“ helps us to understand this close link between obesity, diabetes, hypertension, and renal dysfunction. An elevated body mass index has shown to be one of the major determinants of glomerular hyperfiltration that lead to the development of chronic kidney disease. Interestingly, weight loss can lead to attenuation of hyperfiltration in severely obese patients suggesting a possible therapeutic option to combat obesity-related hyperfiltration.

*Conclusions:* Various treatment strategies had been suggested to decrease impact of obesity on kidneys. These are blood pressure controling, inhibition of the renin-angiotensinaldosterone axis, improving glycemic control, improving dyslipidemia, improving protein uriaand lifestyle modifications. Regardless of the numerous pharmacotherapies, the focus should be on the root cause: obesity.

## 1. Context

Obesity, both directly and indirectly, increases the risk for a variety of disease conditions including diabetes, hypertension, liver disease, and certain cancers, which in turn, decreases the overall lifespan in both men and women.

## 2. Evidence acquisition

Directory of Open Access Journals (DOAJ), Google Scholar, PubMed, and Web of Science were searched with keywords relevant to fat, metabolic syndrome, renal disease and kidney protection.

## 3. Results

The kidney has long been pondered by the minds of individuals from various professions. Scientists, artists, and philosophers alike have commented on the kidneys as a pair, as well as the ability of the body to survive with only a single kidney. In the old testament for example, the kidneys were described as organs with “fat that is upon them” and were often burned upon the altar to allow Yahweh to receive his portion (1,2). By virtue of their deep location within the retroperitoneum, the kidneys were often seen as one’s innermost being and self-consciousness. As many ancient cultures had seen obesity as a symbol of opulence and power, it should come to no surprise that these civilizations held the kidneys in such high regard given their close association with fat. However, in modern society obesity not only connotes laziness, but it has also been overwhelmingly proven to be detrimental to our overall health. According to the 2013 statistics from the CDC, more than 30% of adults are obese in United States (3). This is an alarming trend considering that in 1994, not a single state had an average obesity rate that exceeded 22% (4). Though the dawn of the obesity epidemic in the United States is multifactorial, it can certainly be attributed to increased consumption of high calorie foods, decreased physical activity, and the perpetuation of the belief that being overweight or obese is simply normal. Certainly the United States is not alone in this issue as even developing nations have noted similar trends, thus causing many to believe that obesity problem is on the verge of a pandemic (5,6).

Obesity, both directly and indirectly, increases the risk for a variety of disease conditions including diabetes, hypertension, liver disease, and certain cancers, which in turn, decreases the overall lifespan in both men and women (7). Though the cardiovascular risks of obesity are widely acknowledged, less often identified is the relationship between obesity and renal function (8). The concept of the “Metabolic Syndrome” helps us to understand this close link between obesity, diabetes, hypertension, and renal dysfunction. From an epidemiological standpoint, the increasing rates of obesity have been subsequently followed by an increasing prevalence of diabetes (4). Moreover, the prevalence of diabetes has closely mirrored obesity in terms of geographical distribution, further highlighting this association.

The prevalence of chronic kidney disease substantially increases with the more metabolic syndrome risk factors a patient possess (9). In a review by Abrass, there are a number of pathologic links between metabolic syndrome and chronic kidney disease (10). The author highlights the relationship between hyperinsulinemia and modifications within the kidney, including glomerular hypertrophy, mesangial matrix proliferation, and glomerulosclerosis. These changes are thought to be secondary to glomerular hyperfiltration as well as inflammatory mediators from increased adiposity.

Additionally, obesity-related kidney damage has been posited to be due to hyperlipidemia, increased oxidative stress, increased salt intake, and activation of the sympathetic nervous system (11). Certainly patients with metabolic syndrome possess a number of these risk factors; however, obesity has been shown to be one of the most important independent risk factors. In both diabetic and hypertensive patients, an elevated body mass index has shown to be one of the major determinants of glomerular hyperfiltration (11-13). Elevated BMI also contributes to the development of chronic kidney disease in subjects without hypertension or diabetes (14). Interestingly, weight loss can lead to attenuation of hyperfiltration in severely obese patients, suggesting a possible therapeutic option to combat obesity-related hyperfiltration (15).

Oxidative stress secondary to increased adiposity is also thought to be a contributing factor to hyperfiltration. Li *et al. * showed that the increased GFR noted in metabolic syndrome in the swine model was preceded by activation of oxidative stress and inflammation (16). Increased oxidation of low-density lipoprotein, as observed in obese patients, stimulates synthesis of angiotensin II, which consequently increases TGF-B and plasminogen activator inhibitor-1; these inflammatory cytokines propagate glomerular fibrosis and contribute to chronic kidney disease (17).

In obese patients, cardiac output is increased to adequately maintain perfusion pressures of increased tissue mass. However, the amount of nephrons in the adult do not increase with body size, this elevated cardiac output translates into increased renal plasma flow, and in turn, increased perfusion pressure at each individual nephron (12). At the level of a single nephron, hyperfiltration is posited to precede intraglomerular hypertension which can subsequently lead to changes in efferent and afferent arteriole resistance. If these changes are allowed to persist, GFR falls progressively, leading to albuminuria and may even lead to end-stage renal failure in the long term (11).

## 4. Treatment strategies

### 4.1. Blood pressure control

High blood pressure is a well-known risk factor for kidney damage. Hypertension and autonomic activation have been directly associated with hyperfiltration and this effect is even more pronounced in those who are obese (18,19). Okada *et al.* delineated that hyperfiltration worsened with the severity of the hypertension (20). Any patient who is hypertensive should be appropriately managed with individually catered medications and appropriate lifestyle modifications. The recommended blood pressure goal based in JNC-8 is a target systolic and diastolic blood pressure of less than 140 and 90 mmHg, respectively (21).

### 4.2. Inhibition of the renin-angiotensin-aldosterone axis

One class of antihypertensive medications that has been shown to be effective through a multitude of mechanisms is those that inhibit the renin-angiotensin-aldosterone (RAA) axis. Despite the presence of hyperfiltration, normalizing glomerular pressures could slow the rate of renal dysfunction. Within rat models, agents such as ACE inhibitors have been shown to reduce renal damage by inhibiting the RAA axis (22,23). This benefit is due in part by the ability of these medications to reduce efferent arteriole pressure (22-24). Furthermore, a study has displayed that increased activation of the RAA axis is associated with inflammation, oxidative stress, hypertension, and continued worsening of the renal disease (23). Additionally, there have been marked increases in Angiotensin 1 receptors, NADPH Oxidase activity, and NFkB activation in the rodent models not receiving treatment with ACE inhibitors (23-25). Irbesartan, an angiotensin receptor blocker, was shown to reduce endothelial surface damage in rodent models (25). A further benefit of inhibition of the RAA axis, as evidenced by various trials including the LIFE, MARVAL, IDNT and RENAAL studies, have shown improvement of renal outcomes (26-29). A meta-analysis by Bakris indicates that as systolic blood pressure is lowered we witness a reduction in the rate of decline of the glomerular filtration rate (30). The Ramipril Efficacy in Nephropathy (REIN) study has shown that as serum phosphate increases, the renoprotective effects of ACE inhibitors declines; thus serum phosphate levels should be monitored if there is considerable suspicion that the therapeutic effect is being jeopardized (31).

### 4.3. Improve glycemic control

From a renal standpoint, patients with diabetes have also been shown to benefit from proper glycemic management. Uncontrolled blood sugars are proportionately related to the severity of hyperfiltration. When Okada* et al.* analyzed pre-diabetics, stage 2 pre-diabetics, and diabetics they found a progressive increase in the risk of hyperfiltration that corresponded to the severity of the diabetes (20). In a separate study, impaired fasting levels of glucose was directly correlated with hyperfiltration having accounted for confounding factors including age, sex, smoking status, body mass index, blood pressure, and insulin levels (32).

### 4.4. Improve dyslipidemia

Epidemiologic evidence based on Helsinki heart study and physicians’ health study showed that a higher LDL/HDL ratio is accompanied with a higher rate of decline in kidney function (33,34). Several studies have shown that management of dyslipidemia has improved cardiac and stroke outcomes in those with moderate risk factors and diabetes (35,36). In addition to dietary and exercise regimens, the statins have emerged as a class of medications that have shown to improve glomerular filtration. The Greace trial delineated that atorvastatin use led to increases in creatinine clearance by 12% (37). A controlled, prospective study by Bianchi* et al.* showed that statins are able to decrease proteinuria and preserve kidney function (38). Several small studies have shown that statin therapy improves the cardiovascular risk profile of persons with ESRD (39-41); however, others have detected no mortality benefit from Atorvastatin in type II diabetic dialysis patients with ESRD despite a median 42% reduction in LDL-C (42). Although, there is inconclusive evidence that use of statins in dialysis patients results in cardiovascular benefits, using statins is still a cornerstone of treatment in obesity and dyslipidemia.

### 4.5. Improve proteinuria

Proteinuria is a common finding in obesity. The glomerulopathy seen in obesity is pathologically similar to idiopathic focal and segmental glomerulosclerosis (FSGS), but the former has a more indolent course. The attempt to control and reduce proteinuria is still one of the goals of treatment in obese patients, although, clinical manifestation and podocyte injury is less frequent in the obese population (43). Multiple studies have been shown that ACE inhibitors and ARBs in higher doses are able to reduce proteinuria as well as control high blood pressure (44,45). Aldosterone antagonists are able to decrease proteinuria and might be a good choice in the setting of obesity (46).

### 4.6. A potentially novel treatment

A newer medication that may find a place in the treatment of hyperfiltration is acetazolamide. Current clinical trials are comparing the use of furosemide with acetazolamide to investigate if prior results, which showed an 18% reduction in glomerular hyperfiltration, were due to acetazolamide or a generalized diuretic effect (47,48). The proposed mechanism of acetazolamide’s efficacy is through increased solute delivery to the macula densa, resulting in increased tubuloglomerular feedback, thus inhibiting glomerular filtration (47).

### 4.7. Lifestyle modifications

Regardless of the numerous pharmacotherapies that exist to combat and slow down the progression of renal disease, the focus should be on the root cause: obesity. In the obese individual, the body has established a new pathological set-point. Thus, reversal of this progression is best approached through weight loss, healthier meals, and exercise that will help the body revert out of this state. This approach should be an integral part of the treatment plan for every individual (13). The entire cascade of hyperperfusion and hyperfiltration within the kidney in an obese individual can be reversed by the simple intervention of weight loss. Moreover, weight loss has the added benefit of reducing blood pressure and glucose in patients with diabetes and blood pressure (10).

Per clinical guidelines, patients who are overweight or obese should begin a regimen with an initial goal of 10% weight loss. Diet and exercise play a key role in achieving this goal. It is recommended that each individual exercise for 30 minutes per day at least 5 days per week (49). Dietary goals are equally important, but should be catered to each patient’s baseline and individual needs. General guidelines encourage a low-calorie diet, which expects to reduce intake by 500 to 1,000 calories per day. Additionally, individuals should not only reduce saturated fat intake, but also grossly reduce total caloric intake of fats to 30% or less per day. Maintenance of weight loss is important, thus continued carbohydrate and fat restrictions must be followed (49). As noted by several investigations, salt intake has been shown to increase glomerular hyperfiltration, thus progressive restriction towards 100 mmol (2.3 gram) and eventually 50 mmol (1.2 grams) of salt is recommended; additionally adherence to the DASH diet is advised (22,50-52). Finally, other important lifestyle changes may not be as easily recognized, but nonetheless, they can help patients with glomerular hyperfiltration. These include reducing alcohol consumption, reducing caffeine intake and discontinuing smoking (13).

## 5. Conclusions

Obesity-related renal injury has been posited to be due to hyperlipidemia, increased oxidative stress, increased salt intake, and activation of the sympathetic nervous system. Various treatment strategies had been suggested to decrease impact of obesity on kidneys. These are blood pressure controling, inhibition of the renin-angiotensin-aldosterone axis, improving glycemic control, improving dyslipidemia, improving proteinuria and lifestyle modifications.

## Authors’ contributions

All authors wrote the manuscript equally.

## Conflict of interests

The author declared no competing interests.

## Funding/Support

This research received no specific grant from any funding agency in the public, commercial, or not-for-profit sectors

## References

[1] Bate J. A new and literal translation, from the Hebrew, of the Pentateuch of Moses, and of the historical books of the Old Testament, to the end of the second book of Kings: with notes critical and explanatory. Printed for W. Faden, B. Law, 1773.

[2] Eknoyan G (2005). The kidneys in the Bible: what happened?. J Am Soc Nephrol.

[3] Ogden C, Carroll M, Kit B, Flegal K (2012). Prevalence of obesity in the United States, 2009-2010. NCHS Data Brief.

[4] CDC VitalSigns - Adult Obesity. (n.d.). Retrieved March 6, 2014, from http://www.cdc.gov/VitalSigns/adultobesity/

[5] Popkin BM, Adair LS, Ng SW (2012). Global nutrition transition and the pandemic of obesity in developing countries. Nutr Rev.

[6] Gupta N, Goel K, Shah P, Misra A (2012). Childhood obesity in developing countries: epidemiology, determinants, and prevention. Endocr Rev.

[7] Muennig P, Lubetkin E, Jia H, Franks P (2006). Gender and the Burden of Disease Attributable to Obesity. Am J Public Health.

[8] Lakka HM, Laaksonen DE, Lakka TA, Niskanen LK, Kumpusalo E, Tuomilehto J (2002). The metabolic syndrome and total and cardiovascular disease mortality in middle-aged men. JAMA.

[9] Chen J, Muntner P, Hamm LL, Jones DW, Batuman V, Fonseca V (2004). The metabolic syndrome and chronic kidney disease in USadults. Ann Intern Med.

[10] Abrass CK (2004). Overview: obesity: what does it have to do with kidney disease?. J Am Soc Nephrol.

[11] Palatini P (2012). Glomerular hyperfiltration: a marker of early renal damage in pre-diabetes and pre-hypertension. Nephrol Dial Transplant.

[12] Wuerzner G, Pruijm M, Maillard M, Bovet P, Renaud C, Burnier M (2012). Marked association between obesity and glomerular hyperfiltration: a cross-sectional study in an African population. Am J Kidney Dis.

[13] Zoccali C (2009). Overweight, obesity and metabolic alterations in chronic kidney disease. Prilozi.

[14] Tomaszewski M, Charchar FJ, Maric C, McClure J, Crawford L, Grzeszczak W (2007). Glomerular hyperfiltration: a new marker of metabolic risk. Kidney Int.

[15] Chagnac A, Weinstein T, Herman M, Hirsh J, Gafter U, Ori Y (2003). The effects of weight loss on renal function in patients with severe obesity. J Am Soc Nephrol.

[16] Li Z, Woollard JR, Wang S, Korsmo MJ, Ebrahimi B, Grande JP (2011). Increased glomerular filtration rate in early metabolic syndrome is associated with renal adiposity and microvascular proliferation. Am J Physiol Renal Physiol.

[17] Chalmers L, Kaskel FJ, Bamgbola O (2006). The role of obesity and its bioclinical correlates in the progression of chronic kidney disease. Adv Chronic Kidney Dis.

[18] Schmieder RE, Veelken R, Schobel H, Dominiak P, Mann JF, Luft FC (1997). Glomerular hyperfiltration during sympathetic nervous system activation in early essential hypertension. J Am Soc Nephrol.

[19] Palatini P, Mormino P, Dorigatti F, Santonastaso M, Mos L, De Toni R (2006). Glomerular hyperfiltration predicts the development of microalbuminuria in stage 1 hypertension: the HARVEST. Kidney Int.

[20] Okada R, Yasuda Y, Tsushita K, Wakai K, Hamajima N, Matsuo S (2012). Glomerular hyperfiltration in prediabetes and prehypertension. Nephrol Dial Transplant.

[21] James PA, Oparil S, Carter BL, Cushman WC, Dennison-Himmelfarb C, Handler J 2014 evidence-based guideline for the management of high blood pressure in adults: Report from the panel members appointed to the eighth joint national committee (jnc 8). JAMA.

[22] Taal MW, Brenner BM (2000). Renoprotective benefits of RAS inhibition: from ACEI to angiotensin II antagonists. Kidney Int.

[23] Vaziri ND, Bai Y, Ni Z, Quiroz Y, Pandian R, Rodriguez-Iturbe B (2007). Intra-renal angiotensin II/AT1 receptor, oxidative stress, inflammation, and progressive injury in renal mass reduction. J Pharmacol Exp Ther.

[24] Anderson S, Rennke HG, Brenner BM (1986). Therapeutic advantage of converting enzyme inhibitors in arresting progressive renal disease associated with systemic hypertension in the rat. J Clin Invest.

[25] Kuwabara A, Satoh M, Tomita N, Sasaki T, Kashihara N (2010). Deterioration of glomerular endothelial surface layer induced by oxidative stress is implicated in altered permeability of macromolecules in Zucker fatty rats. Diabetologia.

[26] Brenner BM, Cooper ME, de Zeeuw D, Keane WF, Mitch WE, Parving HH (2001). Effects of losartan on renal and cardiovascular outcomes in patients with type 2 diabetes and nephropathy. N Engl J Med.

[27] Lewis EJ, Hunsicker LG, Clarke WR, Berl T, Pohl MA, Lewis JB (2001). Renoprotective effect of the angiotensin-receptor antagonist irbesartan in patients with nephropathy due to type 2 diabetes. N Engl J Med.

[28] Lindholm LH, Ibsen H, Dahlöf B, Devereux RB, Beevers G, de Faire U (2002). Cardiovascular morbidity and mortality in patients with diabetes in the Losartan Intervention For Endpoint reduction in hypertension study (LIFE): a randomised trial against atenolol. Lancet.

[29] Viberti G, Wheeldon NM (2002). Microalbuminuria reduction with valsartan in patients with type 2 diabetes mellitus: a blood pressure-independent effect. Circulation.

[30] Bakris GL, Williams M, Dworkin L, Elliott WJ, Epstein M, Toto R (2000). Preserving renal function in adults with hypertension and diabetes: a consensus approachNational Kidney Foundation Hypertension and Diabetes Executive Committees Working Group. Am J Kidney Dis.

[31] Zoccali C, Ruggenenti P, Perna A, Leonardis D, Tripepi R, Tripepi G (2011). Phosphate may promote CKD progression and attenuate renoprotective effect of ACE inhibition. J Am Soc Nephrol.

[32] Melsom T, Mathisen UD, Ingebretsen OC, Jenssen TG, Njølstad I, Solbu MD (2011). Impaired fasting glucose is associated with renal hyperfiltration in the general population. Diabetes Care.

[33] Mänttäri M, Tiula E, Alikoski T, Manninen V (1995). Effects of hypertension and dyslipidemia on the decline in renal function. Hypertension.

[34] Schaeffner ES, Kurth T, Curhan GC, Glynn RJ, Rexrode KM, Baigent C (2003). Cholesterol and the risk of renal dysfunction in apparently healthy men. J Am Soc Nephrol.

[35] Colhoun HM, Betteridge DJ, Durrington PN, Hitman GA, Neil HAW, Livingstone SJ (2004). Primary prevention of cardiovascular disease with atorvastatin in type 2 diabetes in the Collaborative Atorvastatin Diabetes Study (CARDS): multicentrerandomised placebo-controlled trial. Lancet.

[36] Sever PS, Dahlöf B, Poulter NR, Wedel H, Beevers G, Caulfield M (2003). Prevention of coronary and stroke events with atorvastatin in hypertensive patients who have average or lower-than-average cholesterol concentrations, in the Anglo-Scandinavian Cardiac Outcomes Trial--Lipid Lowering Arm (ASCOT-LLA): a multicentrerandomised controlled trial. Lancet.

[37] Athyros VG, Mikhailidis DP, Papageorgiou AA, Symeonidis AN, Pehlivanidis AN, Bouloukos VI (2004). The effect of statins versus untreated dyslipidaemia on renal function in patients with coronary heart diseaseA subgroup analysis of the Greek atorvastatin and coronary heart disease evaluation (GREACE) study. J Clin Pathol.

[38] Bianchi S, Bigazzi R, Caiazza A, Campese VM (2003). A controlled, prospective study of the effects of atorvastatin on proteinuria and progression of kidney disease. Am J Kid Dis.

[39] Chang JW, Yang WS, Min WK, Lee SK, Park JS, Kim Kim, SB SB (2002). Effects of simvastatin on high-sensitivity C-reactive protein and serum albumin in hemodialysis patients. J Am Soc Nephrol.

[40] Ikejiri A, Hirano T, Murayama S, Yoshino G, Gushiken N, Hyodo T (2004). Effects of atorvastatin on triglyceride-rich lipoproteins, low-density lipoprotein subclass, and C-reactive protein in hemodialysis patients. Metabolism.

[41] Van den Akker JM, Bredie SJH, Diepenveen SHA, van Tits LJH, Stalenhoef AFH, van Leusen R (2003). Atorvastatin and simvastatin in patients on hemodialysis: effects on lipoproteins, C-reactive protein and in vivo oxidized LDL. J Nephrol.

[42] Wanner C, Krane V, März W, Olschewski M, Mann JFE, Ruf G (2005). Atorvastatin in patients with type 2 diabetes mellitus undergoing hemodialysis. N Engl J Med.

[43] Kambham N, Markowitz GS, Valeri AM, Lin J, D’Agati VD (2001). Obesity-related glomerulopathy: an emerging epidemic. Kidney Int.

[44] Palla R, Panichi V, Finato V, Parrini M Andreini B, Bianchi AM (1994). Effect of increasing doses of lisinopril on proteinuria of normotensive patients with IgA nephropathy and normal renal function. Int J Clin Pharmacol Res.

[45] Parving HH, Lehnert H, Bröchner-Mortensen J, Gomis R, Andersen S, Arner P (2001). The effect of irbesartan on the development of diabetic nephropathy in patients with type 2 diabetes. N Engl J Med.

[46] Bianchi S, Bigazzi R, Campese VM (2006). Long-term effects of spironolactone on proteinuria and kidney function in patients with chronic kidney disease. Kidney Int.

[47] Skøtt P, Hommel E, Bruun NE, Arnold-Larsen S, Parving HH (1998). Effects of acetazolamide on kidney function in type 1 (insulin-dependent) diabetic patients with diabetic nephropathy. Diabetologia.

[48] Effect of Acetazolamide and Furosemide on Obesity-induced Glomerular Hyperfiltration. June 16, 2010. On-going trial http://clinicaltrials.gov/ct2/show/NCT01146288

[49] Clinical guidelines on the identification, evaluation, and treatment of overweight and obesity in adults: executive summary. Expert Panel on the Identification, Evaluation, and Treatment of Overweight in Adults. Am J Clin Nutr 1998;68(4):899-917. 10.1093/ajcn/68.4.899 9771869

[50] Mallamaci F, Leonardis D, Bellizzi V, Zoccali C (1996). Does high salt intake cause hyperfiltration in patients with essential hypertension?. J Hum Hypertens.

[51] Campese VM, Parise M, Karubian F, Bigazzi R (1991). Abnormal renal hemodynamics in black salt-sensitive patients with hypertension. Hypertension.

[52] Svetkey LP, Sacks FM, Obarzanek E, Vollmer WM, Appel LJ, Lin PH (1999). The DASH Diet, Sodium Intake and Blood Pressure Trial (DASH-sodium): rationale and design DASH-Sodium Collaborative Research Group. J Am Diet Assoc.

